# ﻿Two new species of Cicadellinae (Hemiptera, Cicadellidae) from Yunnan, China

**DOI:** 10.3897/zookeys.1259.161557

**Published:** 2025-11-12

**Authors:** Yan Jiang, Xiao-Fei Yu, Mao-Fa Yang

**Affiliations:** 1 Department of Pharmacy, Guizhou Provincial Engineering Research Center of Medical Resourceful Healthcare Products, Guiyang Healthcare Vocational University, Guiyang 550000, China; 2 Institute of Entomology, Guizhou Key Laboratory of Agricultural Biosecurity, Guizhou University, Guiyang 550025, China; 3 College of Tobacco Sciences, Guizhou University, Guiyang 550025, China

**Keywords:** Auchenorrhyncha, China, leafhopper, morphology, taxonomy

## Abstract

Two new species of the subfamily Cicadellinae (Hemiptera: Cicadellidae), *Atkinsoniella piscioscillum* Jiang & Yang, **sp. nov.**, and *A. hippocampus* Jiang & Yang, **sp. nov.** from Yunnan, China are described and illustrated. *Atkinsoniella piscioscillum* Jiang & Yang, **sp. nov.** is similar to *A. yani* Yang, Meng & Li, 2017 in appearance, but can be distinguished by the color of the legs, the characteristic of the two longitudinal red stripes on the corium, and the morphology of the male genitalia. *Atkinsoniella hippocampus* Jiang & Yang, **sp. nov.** is similar to nine *Atkinsoniella* species, but can be differentiated by the characteristics of aedeagus and subgenital plate. All type specimens of the two new species are deposited at the Institute of Entomology, Guizhou University, Guiyang, China (GUGC).

## ﻿Introduction

Cicadellinae is one of the largest subfamilies of Cicadellidae; it includes 341 genera worldwide with 2669 species (Young 1968; Linnavuori and Delong 1977; Feng and Zhang 2017; Yang et al. 2017; Naveed and Zhang 2018; Dmitriev et al. 2022 [onward]). Certain Cicadellinae species have notable economic importance. They infect the xylem of woody and herbaceous plants and spread phytopathogens like bacteria and viruses to crops, ornamental plants, and weeds (Hopkins and Purcell 2002; Redak et al. 2004; Krugner et al. 2019; Kleina et al. 2020). In China, 23 genera and 268 species of Cicadellinae have been recorded (Feng and Zhang 2017; Yang et al. 2017; Jiang et al. 2022, 2023, 2024). Among them, 149 species are distributed in Yunnan Province, accounting for 55.60% of the total number recorded in China. Of these, 69 species are exclusively found in Yunnan Province.

The genus *Atkinsoniella* Distant, 1908 represents a key component of the subfamily Cicadellinae. To date, 105 valid *Atkinsoniella* species have been described worldwide. Of these, 95 species are recorded in China, distributed across 20 provincial-level administrative regions, with Yunnan Province harboring 73 species (Feng and Zhang 2015; Yang et al. 2017; Naveed and Zhang 2018; Jiang et al. 2022, 2023, 2024).

As a part of the Yunnan-Guizhou Plateau, Yunnan is rich in mountains and diverse natural landscapes. It has exhibits dramatic elevation variations, ranging from its lowest point at 76.4 meters to highest peak at 6740 meters (https://www.yn.gov.cn/yngk/gk/201904/t20190403_96255.html). The climate here is complex and diverse, with characteristics of a monsoon climate, a low-latitude climate, and a plateau climate (https://www.yn.gov.cn/yngk/gk/201904/t20190403_96257.html). The unique geographical and climate environment supports a remarkable diversity of plant and animal species. This positions Yunnan as a key region for undocumented Cicadellinae diversity. According to the literature and the specimens we collected, the altitude of Cicadellinae species distributed in Yunnan Province ranges from 270 m to 3500 m (Yang et al. 2017; Jiang et al. 2024). Even so, there are still many regions with various elevational gradients that remain undersampled. In this study, the descriptions, male genitalia, female genitalia, and habitus photographs of two new species, *A. piscioscillum* Jiang & Yang, sp. nov. and *A. hippocampus* Jiang & Yang, sp. nov. from Yunnan Province, China are provided.

## ﻿Material and methods

The specimens were collected by sweeping on shrubs and weeds using insect sweep nets during daylight and by using a 500W high-pressure mercury lamps in the evening; all specimens were preserved in absolute ethanol and stored at -20 °C in the laboratory. The abdomens of specimens were detached and soaked in 10% NaOH solution, boiled for approximately 3 min until the specimens became transparent, rinsed with clean water 2 times to remove residual NaOH solution, then transferred to a slide with glycerol for further dissection, photography and finally preserved in 200 μl PCR tube with glycerol. Habitus and male genitalia were photographed using a KEYENCE VHX-6000 digital microscope and a Nikon Eclipse Ni-E microscope, respectively. Photos were edited and compiled using Adobe Photoshop 2020 software. The length of the body was measured from the vertex to the rear of the forewings using a KEYENCE VHX-6000 digital microscope. The morphological terminology follows Young (1968, 1986) and Yang et al. (2017). The holotype and paratypes were deposited at the Institute of Entomology, Guizhou University, Guiyang, China (GUGC).

## ﻿Taxonomy

### ﻿Genus Atkinsoniella

Taxon classificationAnimaliaHemipteraCicadellidae

Distant, 1908

7B31C833-DB7C-54CA-8623-8BDB7A54D8E6

Atkinsoniella Distant, 1908: 235.
Soibanga Distant, 1908: 236.
Curvufacies Kuoh, 1993: 38.

#### Type species.

*Atkinsoniella decisa* Distant, 1908.

#### Distribution.

Palearctic, Oriental.

### ﻿ Atkinsoniella piscioscillum

Taxon classificationAnimaliaHemipteraCicadellidae

Jiang & Yang sp. nov.

0F3112C5-A421-5032-BB25-0A2813834CD7

https://zoobank.org/F5EC8862-8A15-4D72-8A5E-F0E0BDEA5DA7

**Figure 1.**
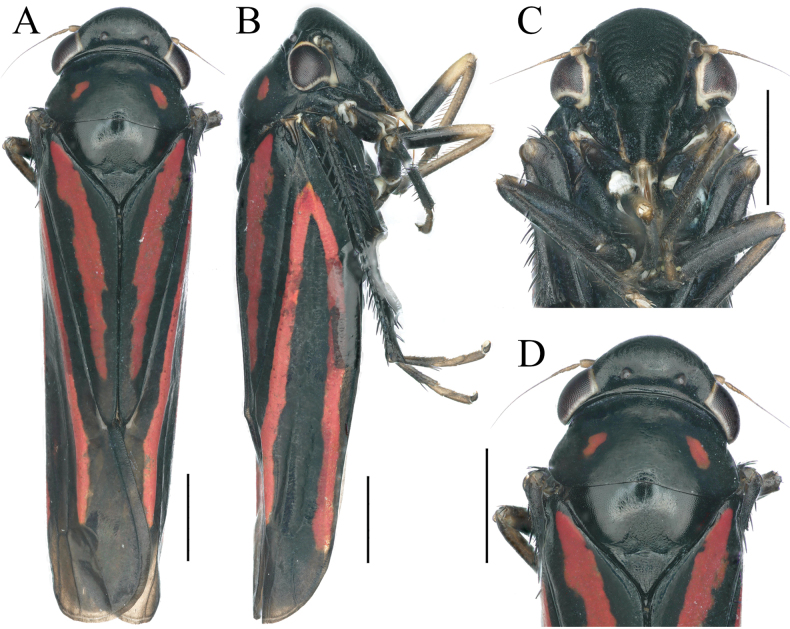
External features of *Atkinsoniella piscioscillum* Jiang & Yang, sp. nov., male holotype. A. Habitus, dorsal view; B. Habitus, lateral view; C. Face, anterior view; D. Head and pronotum, dorsal view. Scale bars: 1000 μm.

**Figure 2.**
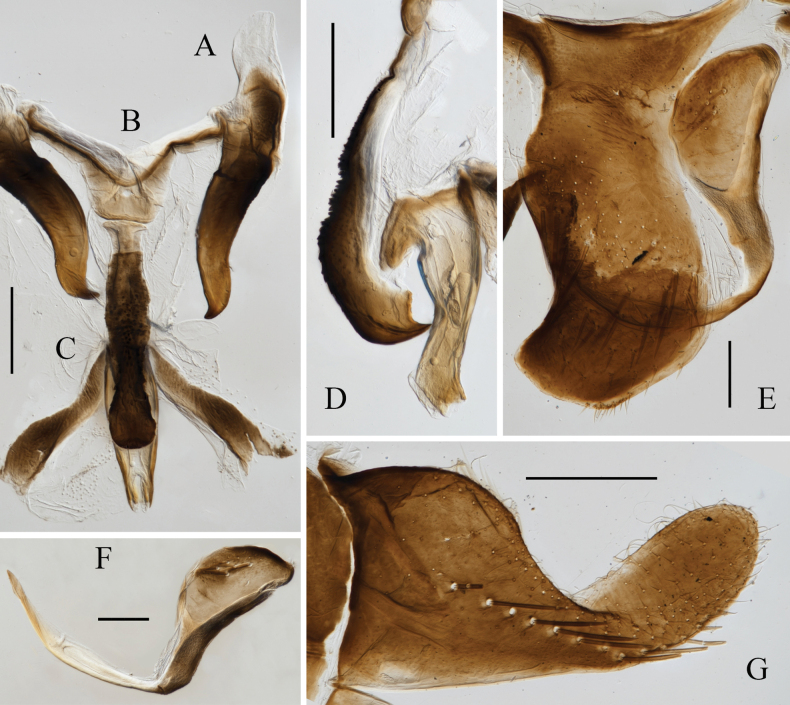
Male genitalia of *Atkinsoniella piscioscillum* Jiang & Yang, sp. nov. A. Style; B. Connective; C. Aedeagus and paraphysis, ventral view; D. Aedeagus and paraphysis, lateral view; E. Pygofer, lateral view; F. Pygofer process; G. subgenital plate, ventral view. Scale bars: 200 μm.

**Figure 3.**
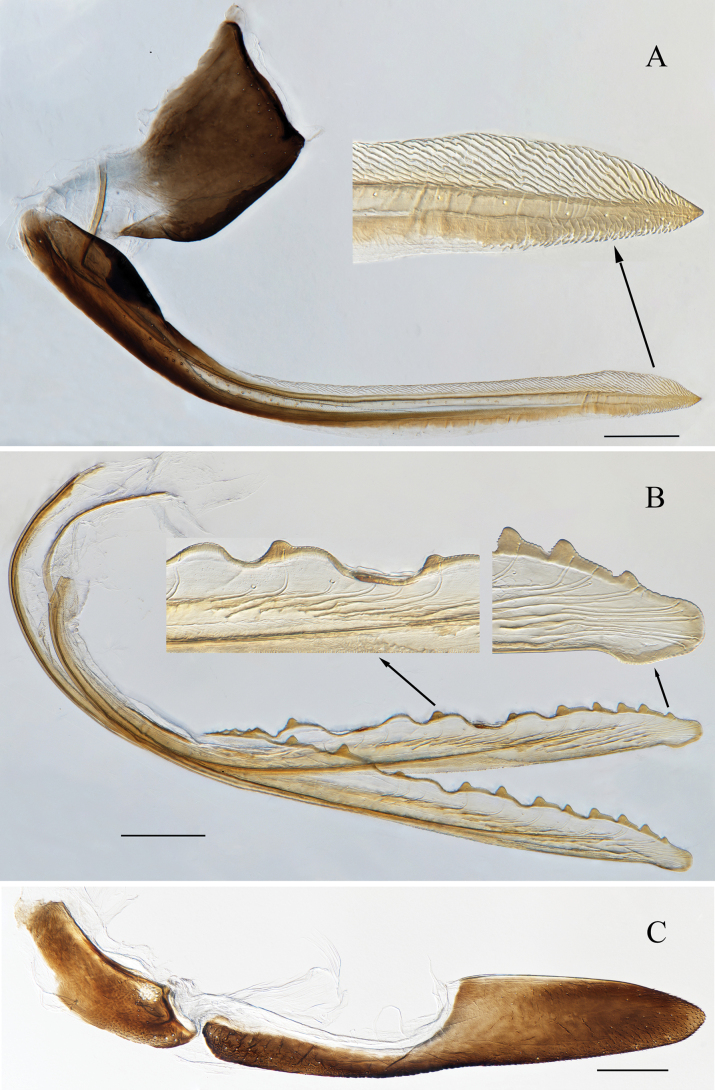
Female genitalia of *Atkinsoniella piscioscillum* Jiang & Yang, sp. nov. A. First valvifer and first valvula, lateral view; B. Second valvula, lateral view; C. Second valvifer and third valvula, lateral view. Scale bars: 200 μm.

Figs 1A–D, 2A–G, 3A–C

#### Description.

Head, thorax, and forewings black. Eyes dark brown with yellowish-white margins, ocelli grayish-white. Pronotum with one oblique posterolateral red stripe on each side. Forewings with three longitudinal red stripes, one extending through clavus, two on corium and converging basally. Face, thorax and abdomen in ventral view, and legs black.

Crown with anterior margin rounded and convex, lateral area of ocelli concave; ocelli located at imaginary line between anterior eye angles and tip of lateral clypeal suture, each ocellus further from the other one than to the adjacent eye. Pronotum wider than head, anterior margin convex arcuately, posterior margin slightly concave medially. Scutellum with transverse depression wavy and slightly posterior to the median. Forewings with apical membranous area not obvious, base of the second and third cells almost aligned transversely. Face with frontoclypeus flat in the median, muscle impressions and clypeal sulcus distinct.

Male pygofer with dorsal and ventral margins nearly parallel, tip bent dorsally and posterior margin truncated obliquely, subapical portion with scattered macrosetae, apex with microsetae; pygofer process with long, moderately thick setae in the center of base, bent dorsad medially, then tip curved anterior dorsally, apex acute and not exceeding dorsal margin of pygofer. Subgenital plate broad at base, posterior half narrow and bent dorsally, with one uniseriate row of macrosetae obliquely, lateral margin and apical half with scattered microsetae. Aedeagus hooked in lateral view, base broader than posterior half, basal ventral portion lamellate, hooked and articulating with paraphysis, ventral margin angulately concave medially, apex bent dorsally with posterior margin truncated. Paraphysis arcuately curved dorsad, with ventral two transverse grooves and dense granular tubercles medially, tip curved dorsally and fish-mouth shaped, apex tapered and articulating with aedeagus, dorsal margin with angular projection subapically and arcuate concave apically. Connective Y-shaped, with short stem. Style broad at subbase, tip tapered and curved.

Female abdominal sternite VII shorter than wide, posterior margin with median concave; pygofer, in lateral view, produced posteriorly, posterior margin narrowly rounded with macrosetae at posterior portion. First valvifer longer than wide. First valvula apex acute, dorsal area with sculptured striae extending from basal portion of blade to apex, ventral area with sculptured stripe at apical portion of blade. Second valvula ventral preapical margin protruding, posterior portion rounded and convex, blade with 11 continuous large triangular teeth on expanded subapical portion and smaller teeth apically and basally, all large teeth as well as apex of apical blade with denticles, ducts distributed in area of sixth teeth to apex of blade. Second valvifer longer than wide, ventral margin with a cluster of short macrosetae subbasally. Third valvula basal 1/2 narrow and posterior 1/2 distinctly expanded, apex obtuse, with microsetae distributed on apical portion and posterior 1/3 ventral margin of blade.

#### Etymology.

The specific epithet, *piscioscillum*, is a combination of the Latin nouns *piscis* and *oscillum*, referring to the tip of the paraphysis in ventral view, which looks like a fish mouth.

#### Measurement.

Length of male 7.2–7.7 mm, females 7.4–8.1 mm.

#### Material examined.

***Holotype*** • ♂, Tai’an Township, Yulong Naxi Autonomous County, Lijiang City, Yunnan Province, China, 2800 m, 8 August 2021, coll. Yan Jiang. ***Paratypes*** • 1♂2♀♀, same data as holotype • 1♀, Jinhong Mountain, Yulong Naxi Autonomous County, Lijiang City, Yunnan Province, China, 2502 m, 7 August 2021, coll. Yan Jiang • 3♂♂2♀♀, Yongchun Township, Weixi Lisu Autonomous County, Diqing Tibetan Autonomous Prefecture, Yunnan Province, China, 2359 m, 10 August 2021, coll. Yan Jiang • 2♀♀, Tongdian Town, Lanping Bai and Pumi Autonomous County, Nujiang Lisu Autonomous Prefecture, Yunnan Province, China, 3006 m, 11 August 2021, coll. Hong-Li He.

#### Remarks.

This species is similar to *A. yani* Yang, Meng & Li, 2017 in appearance, but can be easily differentiated by the following characteristics: (1) the legs of the new species are black, but the legs of the latter are yellow; (2) the two longitudinal red stripes on the corium of the new species converge basally, but the two longitudinal red stripes of the latter are nearly parallel and not joined at the base; (3) the pygofer of the new species with posterior margin truncated obliquely, but the apex of the pygofer of the latter is bifurcated; and (4) the tip of the paraphysis in the new species is fish-mouth shaped in lateral view, but the tip of the paraphysis in the latter is sharp-teeth shaped.

#### Distribution.

China (Yunnan).

### ﻿ Atkinsoniella hippocampus

Taxon classificationAnimaliaHemipteraCicadellidae

Jiang & Yang sp. nov.

3AECA63B-ED57-59E2-A18D-34D8CB26F61C

https://zoobank.org/C7102D11-8837-4972-AC46-1758444E5F52

Figs 4A–D, 5A–G

#### Description.

Dorsum black. Eyes dark, ocelli grayish-white. Pronotum black. Scutellum black, with apex brown. Forewings basal 1/7–4/7 blood-red, with anterior margin, posterior margin, and claval suture black. Face grayish-white, with frontoclypeus black, and anteclypeus with black triangular spot at base. Thorax black in ventral view, legs grayish-white with tibia of forelegs and pretarsus of all legs black brown. Abdomen black.

**Figure 4.**
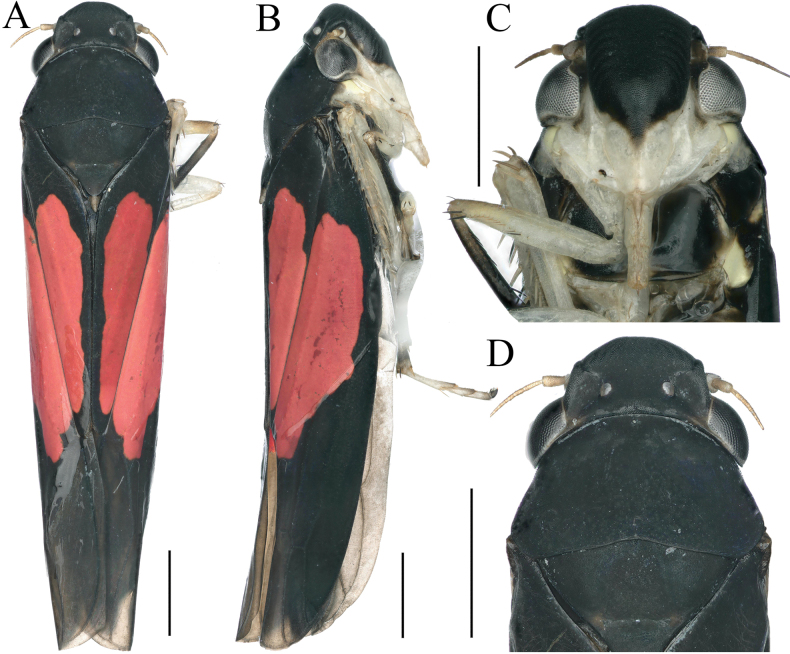
External features of *Atkinsoniella hippocampus* Jiang & Yang, sp. nov., male holotype. A. Habitus, dorsal view; B. Habitus, lateral view; C. Face, anterior view; D. Head and pronotum, dorsal view. Scale bars: 1000 μm.

Crown with anterior margin rounded and convex, median length of the crown nearly equal to 2/3 interocular width, ocelli located at the imaginary line between the anterior eye angles and the tip of the lateral clypeal suture, each ocellus closer to the adjacent eye angle than to another ocellus, lateral area of ocelli concave. Face with frontoclypeus flat medially, muscle impressions distinct, clypeal sulcus distinct in the median, anteclypeus gibbous longitudinally. Pronotum wider than head, anterior margin arcuately convex, posterior margin with triangularly concave medially. Scutellum with transverse depression slightly posterior to the median. Forewings with apical membranous area not obvious, base of second cells more proximal than third cells transversely.

**Figure 5.**
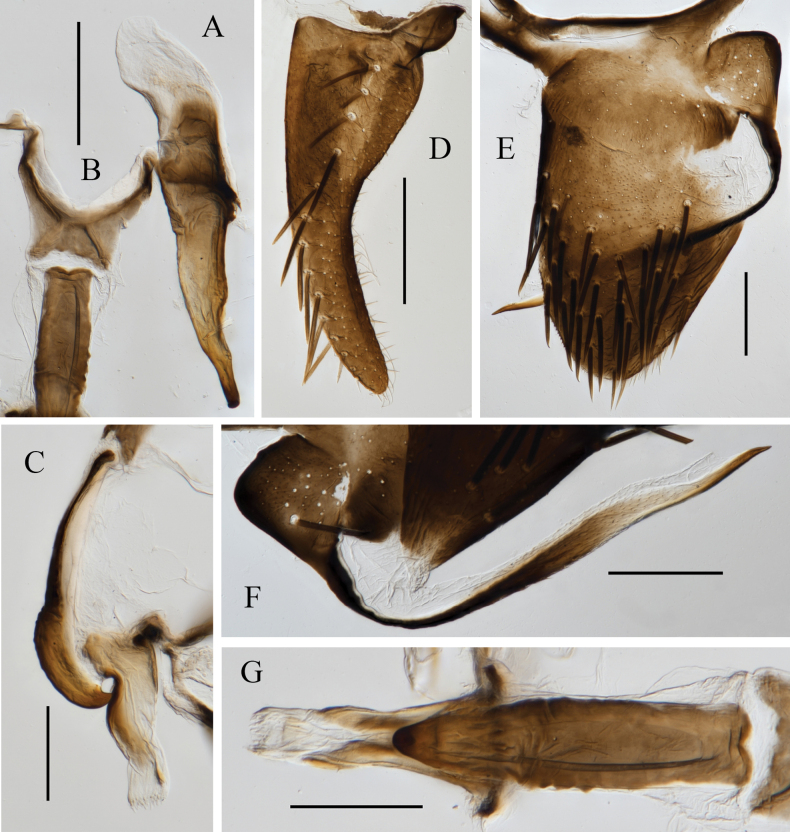
Male genitalia of *Atkinsoniella hippocampus* Jiang & Yang, sp. nov. A. Style; B. Connective; C. Aedeagus and paraphysis, lateral view; D. Subgenital plate, ventral view; E. Pygofer, lateral view; F. Pygofer process; G. Aedeagus and paraphysis, ventral view. Scale bars: 200 μm.

Male pygofer broadly short, narrowly rounded posteriorly, posterior half with macrosetae. Pygofer process slender, base with several macrosetae and microsetae, apical 2/3 bent dorsally and extending straightly and exceeding posterodorsal margin of pygofer, dorsal margin with membranous structure, apex acute, posterior half with microtrichia on surface. Subgenital plate slender, base slightly wider, posterior half gradually narrowed, with a uniseriate row of macrosetae, lateral area of macrosetae with microsetae. Aedeagus hippocampiform in lateral view, ventral margin concave subbasally and subapically, concave of basal ventral margin articulating with paraphysis, dorsal margin concave subapically, base wider than posterior half, apex brush-like. Paraphysis arcuately curved dorsad, ventral margin with transverse striations medially, tip acute, apical half bent dorsad and articulating with aedeagus. Connective Y-shaped, with stem broad and short. Style broad at base, tip tapered, apex shallowly hooked.

#### Etymology.

The specific epithet is from the Greek, *hippocampus*, referring to the hippocampus-shaped aedeagus.

#### Measurement.

Length of male 7.5 mm.

#### Material examined.

***Holotype*** • ♂, Ailao Mountain National Nature Reserve, Xinping County, Yuxi City, Yunnan Province, China, 5 June 2019, coll. Tie-long Xu.

#### Remarks.

This species is similar to *A. biundulata* Meng, Yang & Ni, 2010, *A. longiaurita* Yang, Meng & Li, 2017, *A. atrata* Yang, Meng & Li, 2017, *A. recta* Yang, Meng & Li, 2017, *A. rectangulata* Yang, Meng & Li, 2017, *A. longa* Yang, Meng & Li, 2017, *A. membrana* Yang, Meng & Li, 2017, *A. expanda* Yang, Meng & Li, 2017, and *A. xinfengi* Yang, Meng & Li, 2017, in appearance, but can be easily differentiated by the following special characteristics: (1) aedeagus hippocampiform in lateral view, ventral margin concave subbasally and subapically, dorsal margin concave subapically, base wider than posterior half, apex brush-like; and (2) subgenital plate slender.

#### Distribution.

China (Yunnan).

## Supplementary Material

XML Treatment for Atkinsoniella

XML Treatment for Atkinsoniella piscioscillum

XML Treatment for Atkinsoniella hippocampus

## ﻿Additional information

### Use of AI

No use of AI was reported.

### Author ORCIDs

Yan Jiang https://orcid.org/0000-0002-8980-5344

Xiao-Fei Yu https://orcid.org/0000-0002-1362-9524

Mao-Fa Yang https://orcid.org/0000-0002-5523-6825

### Data availability

All of the data that support the findings of this study are available in the main text.
